# Social stratification in networks: insights from co-authorship networks

**DOI:** 10.1098/rsif.2022.0555

**Published:** 2023-01-04

**Authors:** Zeinab S. Jalali, Josh Introne, Sucheta Soundarajan

**Affiliations:** ^1^ Electrical Engineering and Computer Science, Syracuse University, NY, Syracuse, USA; ^2^ School of Information Studies, Syracuse University, NY, Syracuse, USA

**Keywords:** social stratification, social networks, class stratification

## Abstract

It has been observed that real-world social networks often exhibit stratification along economic or other lines, with consequences for class mobility and access to opportunities. With the rise in human interaction data and extensive use of online social networks, the structure of social networks (representing connections between individuals) can be used for measuring stratification. However, although stratification has been studied extensively in the social sciences, there is no single, generally applicable metric for measuring the level of stratification in a network. In this work, we first propose the novel Stratification Assortativity (StA) metric, which measures the extent to which a network is stratified into different tiers. Then, we use the StA metric to perform an in-depth analysis of the stratification of five co-authorship networks. We examine the evolution of these networks over 50 years and show that these fields demonstrate an increasing level of stratification over time, and, correspondingly, the trajectory of a researcher’s career is increasingly correlated with her entry point into the network.

## 1. Introduction

Human social networks play a critical role in the trajectories of people’s lives. A highly desirable property of dynamic societal networks is that they should allow for individuals to rise and fall on the basis of their own merits, rather than their inherited positional inequalities [1]. This basic property has been described many times in the sociological and philosophical literature [2].

However, in real societies, individuals in human networks are commonly divided into a hierarchical arrangement based on different attributes such as importance, wealth, knowledge and power. This phenomenon is known as *social stratification* [3], and stratification along economic or class-based lines has been one of the most important topics of study in the modern social sciences [4]. Social stratification, as well as its counterpart *social mobility*, governs the trajectories of people’s lives, including the extent of prejudice that they face [5], their careers and occupations [6] and the likelihood that they will experience violence [7].

Historically, social science researchers studying stratification have been armed with domain knowledge about the nature of stratification in the system: in particular, knowledge of the classes of interest, such as upper, middle and lower socioeconomic classes). These classes are often defined with respect to occupation, living conditions, socioeconomic status, etc. [8,9]. Existing empirical analysis of stratification tends to either study social mobility as a proxy for stratification or, if network connections are known, examine inter-class connections between predefined classes [10]. However, these methods tend to be ad hoc, relying on *a priori* knowledge about the classes.

Although stratification has been extensively documented (especially in non-network settings), to our knowledge, there is no single metric to quantify the level of stratification in a network and there is a need for an interpretable, quantitative metric to summarize network stratification in a single number. There are several reasons for this. (i) In many cases, one may not know the classes ahead of time. While economic classes are well established, this may not be the case for other domains. (ii) When comparing different ecosystems (e.g. Facebook versus Twitter), it is useful to have a single number in order to perform a quantitative comparison. In the long run, this may lead to discovery of universal laws about stratification.

In the first part of our work, we introduce the Stratification Assortativity (StA) metric, which measures a network’s stratification with respect to an attribute of interest. StA differs from existing assortativity metrics in that it is based on scalar characteristics that give rise to a set of ordered classes, whereas other assortativity metrics are either based on categorical characteristics that divide a network into non-ordered groups or scalar characteristics that do not consider group memberships [11].

In the second part of our work, we apply our proposed metric to understand stratification in scientific co-authorship networks. A fair amount of work has examined collaboration networks with respect to stratification and equality. Most of these studies use centrality metrics to show the correlation between an individual’s position within the collaboration network and their success; and the ramifications of such correlation to stratification and inequality [12,13]. None of them have studied the evolution of co-authorship networks with respect to long-term stratification and the success of researchers.

We perform an in-depth analysis of five co-authorship networks with respect to author *h*-index,^1^ examining the evolution of these networks over 50 years, and demonstrate that networks evolve into a highly stratified state. Using our proposed StA metric, we show that these fields demonstrate high levels of stratification. Interestingly, we also find that while other types of assortativity decrease over time, stratification increases: in other words, while individuals collaborate with a more diverse set of researchers (with respect to *h*-index) over time, stratification actually increases. Moreover, as stratification increases, the trajectory of a researcher’s career becomes increasingly correlated with her entry point into the network.

The major contributions of this work are as follows: (i) we propose the novel StA metric and introduce an algorithm for identifying classes in a stratified network, and (ii) we perform an extensive analysis of stratification in scientific co-authorship networks and show how networks evolve into a highly stratified state as they age, using both topological properties and success score of researchers.

## 2. Background

In this section, we provide background on social stratification in societies and social networks and describe existing research on stratification in collaboration networks.

### 2.1. Social stratification overview

The study of social stratification has been one of the most important topics in modern sociology and economics [4]. While, historically, there has been disagreement on what exactly constitutes a stratified system, there is some consensus that, at the least, a stratified system requires a ranking or hierarchy of people and groups; acknowledgement, acceptance and legitimation of that ranking; and a correlation between one’s position in the ranking and access to power, prestige or resources [14]. Note that social stratification is not synonymous with social inequality: while social inequality can be a cause of social stratification [9], there are societies in which such inequalities have not created the separate classes present in a stratified society [15].

In the Marxian perspective, stratification or class divisions occur due to the division of individuals based on ‘control and ownership of the means of production and labour power’ [14,16]. Weber extended this one-dimensional view of social stratification to multi-dimensions and considered other types of ownership such as skills, status and organizational power [14,17]. These views are in contrast to the functional view of stratification espoused by Durkheim, who distinguished external inequalities (those imposed by society) from internal inequalities (based on personal merits, such as talent), and believed that the latter type are necessary for the functioning of society [14].

Alternative notions of class also exist: for example, Bourdieu suggested that a class is a set of people with similar nature and living conditions [8]. In Bourdieu’s perspective, class structure in a society is a multi-dimensional space which is shaped by the distributions of different forms of social, economic and cultural capital. For Bourdieu, social classes are constructed as social groups, through ‘articulation, representation and mobilization, by relevant parties or unions’ [18,19].

Intrinsically connected to the notion of social stratification is that of social class. When analysing the stratification of a society, it is useful to identify specific social classes. Defined social classes can directly be used to measure social stratification or be used as a basis of other analysis. For instance, some studies have examined social mobility between classes [10]. Social class refers to hierarchical social categories arising from different relationships in the society [20]. These social classes either divide individuals into categories where boundaries are clearly identified [21] or divide individuals into points along a one-dimensional scale [21].

The study of social stratification is closely related to that of social mobility [22], and social mobility (or the lack thereof) is a driving process behind social stratification [23]. It is known that social stratification can influence prejudice [5], social capital [24], probability of victimization [7], occupation [6] and other crucial factors in the lives of individuals.

Many existing measures of social networks can provide evidence of stratification, but we are not aware of any measures that confirm its presence. For instance, aggregate-level social metrics like the Gini coefficient measure inequality [25].

Another well-studied type of inequality is the Matthew effect (the rich-get-richer or preferential attachment phenomenon) [26], and numerous works have explored quantifying the strength of such a phenomenon [27].

With the rise in human interaction data and extensive use of online social networks, the structure of social networks can be used to study social classes and social stratification [9]. For instance, the capability of individuals to be upwardly mobile can be estimated by examining their connections to higher status individuals in networks [28]. In other words, networks provide an opportunity to study the emergence of social stratification [3], which can help to understand how decisions of individuals can lead to a socially stratified network.

Some empirical analyses on networks have examined social mobility as a proxy for stratification or, if network connections are known, individually examine inter-class connections between predefined classes [10]. These methods are closer to the phenomenon of stratification than measures of inequality, but they are nonetheless indirect (inferred from its consequences). Moreover, measuring social mobility requires long-term temporal data, and looking at inter-class connections between predefined classes is not generalizable.

For instance, Hodler *et al.* use an index which measures the probability that for any random pair of individuals, the poorer individual is deprived of opportunities associated with ethnic class boundaries. This metric uses ethnicity as a proxy for social distance and examines connections between individuals in the context of wealth [5]. However, the phenomenon targeted by Hodler’s metric could arise for other reasons (e.g. racism), and it does not explicitly examine the division of the network into social classes.

Various measures of homophily are also related to stratification, but do not distinguish between ordered strata. For example, the scalar sssortativity coefficient (SAC) [11] is like the Matthew effect in that it can reveal a process of preferential attachment, but is better understood as a measure of overall inequality without considering the division of the network into any specific classes. Modularity [29] and the discrete assortativity coefficient (DAC) [11] consider the division of network into classes, but these classes are not necessarily tiered and do not satisfy the social class definitions. Details of these metrics (modularity, discrete/scalar assortativity) are provided in electronic supplementary material, appendix. None of the existing metrics measure the extent to which the network is divided into *ordered classes*. Our first major contribution in this paper is to propose a metric that satisfies this requirement.

### 2.2. Stratification in collaboration networks

The structures of collaboration networks are important for the diffusion and production of scientific knowledge, and can have an impact on the productivity and work done by the researchers [30]. Social connections in these networks affect the career trajectories of researchers [31,32]. Accordingly, the study of stratification and inequality in collaboration networks has attracted recent attention.

Most of these studies use centrality metrics to analyse networks. For instance Yin *et al.* [31] studied COLLNET, a small collaboration network with respect to stratification. This work demonstrated that certain nodes in favoured locations that are densely connected cause inequality or stratification. Abbasi *et al.* and Liu *et al.* study co-authorship networks and show the research performance of researchers in terms of their *h-*index [12,13]. McCarty *et al.* [33] show the importance of the positions of researchers in a co-authorship network by predicting the *h-*index of authors in the future from their current position. Most of the works in this area focus on analysing one snapshot of the network. However, there are some works on analysing the evolution of collaboration networks. For instance, Servia-Rodríguez *et al.* [34] study the academic success of researchers using co-authorship networks over time and show the correlation between centrality and citations.

Other works have studied stratification and inequality in collaboration networks from different perspectives. Some of these works examine the inequality of citations among researchers. Dong *et al.* [35] used the Gini index and quantified inequality in citation impact in different stages of academic life, and showed that the majority of citations come from a small percentage of researchers.

## 3. Proposed metric: stratification assortativity

In this section, we first introduce the problem of measuring social stratification in networks. Then, we propose (StA), an assortativity metric for measuring network social stratification.

### 3.1. Goal

Assume that we are given an undirected, unweighted network/graph *G*(*V*, *E*) with vertices *V* and edges *E* and binary adjacency matrix **A**. Nodes in *G* have a numeric *characteristic attribute* of interest. This characteristic attribute represents a node’s status (e.g. wealth, professional success, etc.), with higher values indicating a higher status. This attribute is used to identify the social class hierarchy in the networks that causes social stratification. (Note that while the attribute is required, specific class boundaries are not.)

*Our goal is to propose an assortativity metric for measuring network social stratification, the division of a network into ordered tiers (social classes) based on the attribute of interest.*

At a high level, a network is socially stratified if nodes can be partitioned into social classes corresponding to contiguous intervals of the attribute value, such that (i) those classes are separated in the graph topology and (ii) individuals tend to connect to others with similar attribute values. This latter requirement suggests that to the extent that inter-class connections exist, they should be between nodes in similar classes. For instance, later in this study we will consider co-authorship networks, where nodes represent authors and edges represent collaborations. In such networks, a reasonable attribute of interest is the *h*-index of authors. Such a network may be considered stratified if nodes separate into e.g. classes (groups) of high, medium and low *h*-indices, and both inter- and intra-class connections tend to be with nodes of similar scores.

#### 3.1.1. Desired properties

The proposed metric should have the following properties:

**Property 3.1.**

The characteristic attribute should be a *scalar characteristic* and the *actual values* of the characteristic attribute should be taken into account. The metric should more greatly reward connections between nodes with very similar attribute scores, and more greatly penalize connections with very different attribute scores. This property ensures that inter-class connections between nodes in very different tiers are penalized more greatly than those in adjacent tiers.

**Property 3.2.**

The scalar characteristic should be tied to a status feature that allows for grouping into multiple, *ordered tiers* (social classes). Categorical properties are not appropriate, unless they can be associated with a numerical status-related property. Separation with respect to such properties is better measured using other assortativity metrics.

**Property 3.3.**

The metric should use *class membership*; these classes can be known/unknown ahead of time. Like other assortativity metrics, it should be based on connections within and between classes, where more connections within the same class and fewer connections between different classes tend to increase the metric value. Note that this property is different from property 3.2, as property 3.2 takes orders into account, while property 3.3 does not (e.g. modularity/DAC are based on class membership, and so satisfy property 3.3, but not property 3.2).

**Property 3.4.**

*Mutual segregation* is needed for stratification of two classes. If, due to differences in class sizes, the fraction of connections inside a class A is much higher than the fraction of connection between A and B, but the fraction of connections within B is not higher than those from B to A, then A and B are not considered highly segregated. For instance, suppose that A is large and B is small, and that there are *n*_1_ intra-class connections inside A, *n*_2_ intra-class connections inside B and *n*_3_ inter-class connections. If *n*_1_ >>*n*_3_, the fraction of intra-class connections is very high. However, this high fraction does not necessarily correspond to a high level of stratification, because while *n*_3_ ≥ *n*_2_, members of B are connected to members A as much as they are connected to each other. Such a network should not be considered highly stratified.

A real-world example of this can be seen in socioeconomic classes where the size of the upper class is much smaller than the middle class and lower class. Whether the upper class is segregated or not has a huge impact on the social stratification of the society.

### 3.2. Measuring stratification assortativity

The StA metric is inspired by DAC and SAC [11], which measure the tendency of nodes in the networks to connect with like-minded others, and is reformulated in a way that captures the desired properties as explained in the previous section. Table 1 shows a summary of desired properties and whether these metrics satisfy them. As we see, the DAC and SAC metrics do not satisfy all the desired properties.

**Table 1.** Summary of desired properties and whether different metrics satisfy them.

metrics	scalar	ordered	class	mutual
	values	tiers	membership	segregation
modularity	no	no	*yes*	no
discrete assortativity	no	no	*yes*	no
scalar assortativity	*yes*	no	no	no
stratification assortativity	*yes*	*yes*	*yes*	*yes*

StA measures stratification in the given network by first defining the network *stratification score* as the average stratification over all classes. It considers a class to be maximally stratified if nodes of the class only have connections to nodes inside the same class, and minimally stratified if nodes of the class only have connections to nodes in the most distant classes. Then, the *stratification score* is compared with the expected *stratification score* of a random graph with similar properties in order to determine whether the observed network structure is statistically surprising. This is based on the idea that if the number of inter-class connections is significantly more or significantly less than what we expect in a random network, then something interesting is happening in the network [29]. Finding the statistical significance of a property by comparing it with a random structure is common in different network science studies [36,37]. Finally, the final score is normalized to have a value between −1 and 1.

#### 3.2.1. Input

(i) An undirected, unweighted graph *G* = (*V*, *E*), where each node *u* has a characteristic attribute value, denoted by *s*(*u*). (Definitions can be easily modified for a weighted graph.)
(ii) *Social similarity function* *w*(*s*_1_, *s*_2_) which measures the distance between characteristic attribute scores *s*_1_ and *s*_2_. In this work, we define *w*(*s*(*u*), *s*(*v*)) = 1 − (|*s*(*u*) − *s*(*v*)|/max(*S*) − min(*S*)), where *S* is the score distribution for all nodes in the network *G* and *u*, *v* ∈ *V*. Depending on the domain, other functions may be more appropriate. These weights are intended to capture the effect of the actual values of node scores (property 3.1).
(iii) If classes are known: *k* classes *C* = {*c*_1_, *c*_2_, …, *c*_*k*_}, where each *c*_*i*_ represents the nodes with scores within a contiguous portion of the range of possible *score* values (property 3.2). Let *c*(*u*) represent the class membership of *u* (i.e. if *s*(*u*) is in the range represented by *c*_*i*_, then *c*(*u*) = *i*) (property 3.3) (in §3.3.1, we explain how to find classes if they are not known ahead of time).

#### 3.2.2. Computation

First, we define a weighted version of graph *G*, where the weight of each edge (*u*, *v*) ∈ *E* is computed by the *social similarity function*. We then define the StA of the weighted network *G* as

$$StA(G)=\frac{S_{\mathrm{strat}}(G)-E(S_{\mathrm{strat}}(G^{\prime }))}{\mathrm{Max}(S_{\mathrm{strat}}(G))-E(S_{\mathrm{strat}}(G^{\prime }))}.$$

Here, *S*_strat_(*G*) is the *stratification score* of the weighted network *G*, *E*(*S*_strat_(*G*′)) is the expected stratification score of a random network *G*′ with the same weighted degree distribution as network *G* and Max(*S*_strat_(*G*)) is the maximum stratification score among all networks with the same density and set of nodes with the same scalar attribute and class membership as nodes in *G*. These values are computed as

$$\begin{array}{rl} & S_{\mathrm{strat}}(G)=\sum_{c_{i}\in C}\sum_{(u,v)\in E}\frac{\mathrm{sim}(u,v,c_{i})}{\mathrm{sim}(u,v,c_{i})+\mathrm{dissim}(u,v,c_{i})},\\ & ES_{\mathrm{strat}}(G)=\sum_{c_{i}\in C}\sum_{(u,v)\in E}\frac{E(\mathrm{sim}(u,v,c_{i}))}{E(\mathrm{sim}(u,v,c_{i}))+E(\mathrm{dissim}(u,v,c_{i}))}\\ \mathrm{and}\; & \mathrm{Max}(S_{\mathrm{strat}}(G))=k, \end{array}$$

where sim(*u*, *v*) and dissim(*u*, *v*) are the similarity and dissimilarity of nodes *u* and *v*, respectively, and *E*(sim(*u*, *v*)) and *E*(dissim(*u*, *v*) are the expected similarity and dissimilarity of nodes *u* and *v* in networks with the same weighted degree distribution as *G*. Details on how these values are defined are provided in electronic supplementary material, appendix.

### 3.3. Properties of StA metric

StA is a real number between −1 and 1, with 1 representing a network that is fully stratified and −1 corresponding to a dis-stratified network and 0 corresponding to a network with balanced inter- versus intra-class connections.

In a fully stratified network with *k* classes, all connections are between nodes of one class (intra-class connections) and there are no connections between nodes of different classes (inter-class connections). In a fully unstratified network, all connections are inter-class connections. StA is greater than 0 if there are more normalized weighted connections between similar nodes from one class than dissimilar nodes from different classes and StA is lower than 0 if there are more normalized weighted connections between dissimilar nodes from different classes than similar nodes from one class.

Next, we explain why StA properly measures stratification, while other assortativity metrics (DAC and SAC) cannot measure it.

For purposes of this discussion, suppose that we have a set of networks with 16 nodes where each node has a merit score (here, the node ID is equal to its score). Suppose nodes are divided into four classes: low-score nodes (nodes 1–4), medium–low-score nodes (nodes 5–8), medium–high-score nodes (nodes 9–12) and high-score nodes (nodes 13–16).

**Figure 1.**
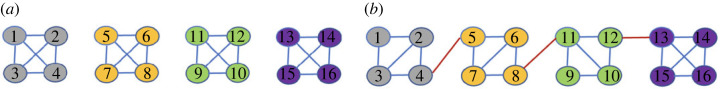
Stratification comparison between *G*_1_ and *G*_2_. Node scores are written inside the nodes. There is no inter-class connections in *G*_1_, thus *G*_1_ is more stratified than *G*_2_ but SAC of *G*_1_ is lower than *G*_2_. (*a*) G1: StA = 1.0, DAC = 1.0, SAC = 0.92 and (*b*) G2: StA = 0.96, DAC = 0.83, SAC = 0.94

Figure 1 shows two networks with these nodes and different topologies. In graph *G*_1_, there are no inter-class connections. As *S*_strat_(*G*) = Max(*S*_strat_(*G*)) when there are no inter-class connections, the StA of *G*_1_ is equal to 1 as expected. In graph *G*_2_, we see three inter-class connections and the StA of *G*_2_ is equal to 0.96 which is lower than *G*_1_, as expected. However, if we compare the SAC of graphs *G*_1_ and *G*_2_, SAC(*G*_1_) < SAC(*G*_2_). This happens because SAC does not consider specific classes (property 3.3) and ordered tiers (property 3.2). In graph *G*_2_, there is a path from nodes in any class to nodes from other classes, whereas in graph *G*_1_, there is no path from nodes of one class to the other classes. Thus, network *G*_1_ is more stratified, as described by StA.

**Figure 2.**
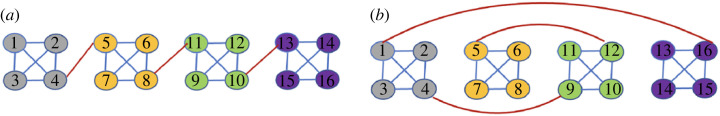
Stratification comparison between *G*_3_ and *G*_4_. Node scores are written inside the nodes. Distance between low-score nodes and high-score nodes in *G*_3_ is higher than *G*_4_, thus *G*_3_ is more stratified than *G*_2_ but DAC of *G*_3_ is equal to *G*_4_. (*a*) G3: StA = 0.96, DAC = 0.85, SAC = 0.91 and (*b*) G4: StA = 0.85, DAC = 0.85, SAC = 0.68.

Next, consider graphs *G*_3_ and *G*_4_ as showed in figure 2. The node properties of these networks are similar to *G*_1_ and *G*_2_. If we compare graphs *G*_3_ and *G*_4_, we see that the number of intra-class connections and inter-class connections are the same. However, inter-class connections in *G*_3_ are edges between similar nodes whereas intra-class connections in *G*_4_ are edges between dissimilar nodes. In other words, the distance between low-score nodes and high-score nodes is higher in *G*_3_ compared with *G*_4_. Thus, we expect StA of *G*_3_ to be higher than *G*_4_. We see that StA(*G*_3_) > StA(*G*_4_) as expected. However, if we compare the DAC of graphs *G*_3_ and *G*_4_, DAC(*G*_3_) = DAC(*G*_4_). This happens because DAC does not consider scalar values (property 3.1) and ordered tiers (property 3.2).

**Figure 3.**
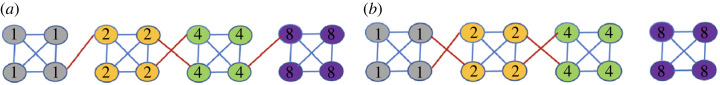
Stratification comparison between *G*_5_ and *G*_6_. Node scores are written inside the nodes. High-score nodes are segregated from the rest of the network in *G*_6_, thus *G*_6_ is more stratified than *G*_5_ but DAC of *G*_6_ is equal to *G*_5_. (*a*) G5: StA = 0.90, DAC = 0.81, SAC = 0.93 and (*b*) G6: StA = 0.93, DAC = 0.81, SAC = 0.97.

Finally, consider graphs *G*_5_ and *G*_6_ as shown in figure 3. The node properties of these networks are similar to *G*_1_–*G*_4_ except that the node number is not equal to its score. Nodes 1–4 have score equal to 1, nodes 5–8 have score equal to 2, nodes 9–12 have score equal to 4 and nodes 13–16 have score equal to 8. If we compare graphs *G*_5_ and *G*_6_, we see that although the number of inter- versus intra-class connections in *G*_5_ and *G*_6_ are the same, high-score class nodes are segregated from the rest of the network in *G*_6_ (there is no connection between high-score nodes and the rest of the network), while in *G*_5_, high-score nodes have access to each other. Thus, we expect the stratification of *G*_6_ to be higher than *G*_5_. We see that StA(*G*_5_) < StA(*G*_6_) as expected. However, if we compare the DAC of graphs *G*_5_ and *G*_6_, DAC(*G*_5_) = DAC(*G*_6_). This happens because DAC does not take mutual segregation into account and is based on the overall inter- versus intra-class connections (property 3.4). Note that we set scores in these networks in a way that inter- and intra-class connections in both networks have the same weight because we wanted to consider class impact and did not want other factors to interfere in the comparison process.

#### 3.3.1. Identifying class boundaries

Depending on the application under study, the social class hierarchy may or may not be available to the researcher. In many real applications, these classes are known ahead of time. For example, it is conventional in economic analysis of Western societies to define a lower, middle and upper class [38]. However, in other networks, such as interactions in meetings or conferences [3], it is possible that neither the class boundaries nor even the number of classes is known ahead of time.

If classes are not given, one must first detect their boundaries. Here, we are interested in social classes that result in the greatest StA. For instance, in a society that is actually divided into lower, middle and upper class, there might be many ways of partitioning the network into non-stratified classes, but the existence of a stratified partition is of great importance.

To find these classes, we use the MaxStrat algorithm. MaxStrat is a dynamic programming-based heuristic that is based on maximizing the non-normalized version of the StA (StA′ = *S*_strat_(*G*) − *E*(*S*_strat_(*G*′)) ). We explain the full details of MaxStrat in electronic supplementary material, appendix.

## 4. Social stratification in collaboration networks

Here, we study the social stratification over time in four co-authorship networks from the fields of Computational Linguistics, Natural Language Processing (NLP), Computational Biology and Biomedical Engineering. These fields have been active for approximately 50 years and were chosen because they are young enough that full data is available (in contrast to very old fields like general physics, where much of the early co-authorship information may not be accessible) but old enough for meaningful evolution to have occurred. This 50-year period covers the bulk of the active period for these fields: before 1966, there were very few papers in these fields, and the dataset is incomplete for papers published after 2015.

### 4.1. Datasets

#### 4.1.1. Graph data

To generate these networks, we extracted papers published in these four fields from the Microsoft Academic Graph (MAG) [39] over the time period from 1966 to 2015.^2^ Each node represents a researcher (author on a paper), and a link between two nodes indicates that they have published a paper together at least once. We treat these networks as undirected and unweighted.^3^

#### 4.1.2. Snapshots

**Figure 4.**
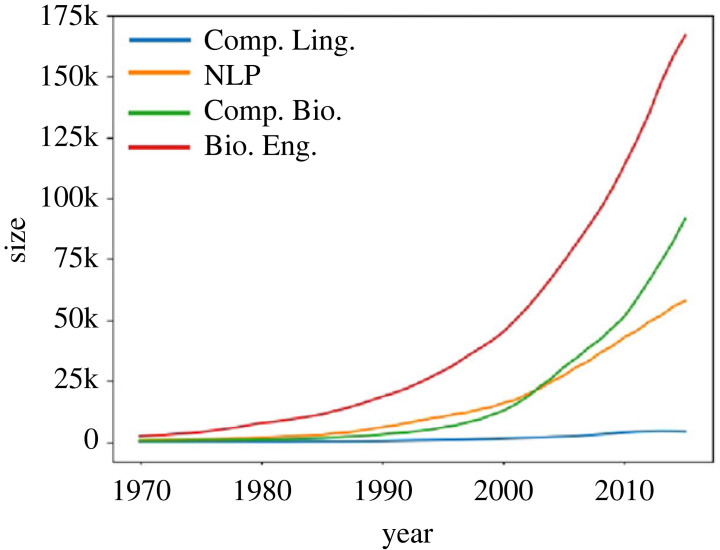
Size of networks (number of nodes).

For each field, we generated 5-year rolling network snapshots spanning 1966–2015 (each snapshot begins 1 year later than the previous). Each field contains at least 11k authors. Table 2 shows dataset statistics and the size of the snapshots is provided in figure 4.

**Table 2.** Dataset statistics.

networks	no. authors	no. connections (distinct)	no. connections (all)
computational linguistics	11k	22k	23k
NLP	137k	398k	476k
computational biology	174k	1.6M	1.7M
biomedical engineering	410k	1.3M	1.4M

#### 4.1.3. Node scores

Node scores are defined by author *h*-indices, computed using citation data within the field up to that year.

### 4.2. Results

In this section, we study stratification in these different fields and explore how stratification changes over time. To perform this analysis, we use the StA metric, which we earlier demonstrated on toy examples.

#### 4.2.1. StA of different fields

Here, we examine the StA of different fields. Our results show that as the network ages, people display a higher tendency to collaborate with members of their same class or nearby classes. In particular, as the field evolves, high-score nodes have a very strong tendency to collaborate with other high-score nodes.

**Figure 5.**
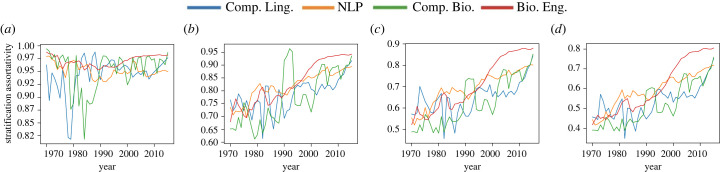
Maximum StA for varying number of classes. (*a*) Two classes, (*b*) three classes, (*c*) four classes and (*d*) five classes.

First, we use the MaxStrat algorithm (defined in electronic supplementary material, appendix) to identify class boundaries for two to five classes. Figure 5 shows the StA of the 5-year snapshots from all four fields. The results demonstrate that all networks have a fairly high level of stratification (above 0.45); and with the exception of the case when we divide into only two classes, StA *increases over time.*

The highest values of StA are obtained with only two classes. To understand why this is so, we examined the *h*-indices that fall into each class. We observed that in almost all snapshots, over half of the nodes have an *h*-index of 0, and only a tiny minority have an *h*-index greater than two (0–13% for Comp. Ling., 1–13% for NLP, 0–7 % for Comp. Bio. and 1–8% for Bio. Eng.); however, because the *h*-indices are integer valued, finer granularity is impossible. The most natural class division, thus, is to put every node with a low *h*-index (0 or 1) into one class, and every other node into a second class. Further stratification within that upper class is outweighted by the stratification between the upper class and lower class.

**Figure 6.**
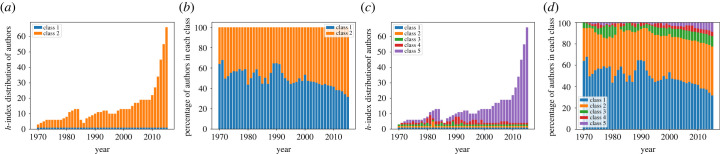
Optimal class boundaries and sizes for two and five classes on computational linguistics networks. (*a*) Two class boundaries, (*b*) fraction of nodes, (*c*) five class boundaries and (*d*) fraction of nodes.

Figure 6 shows optimal boundaries and class sizes for two and five classes for the NLP dataset (we observed similar results in other datasets). In both cases, MaxStrat identifies nodes with score equal to zero as the lowest class for all datasets throughout all years, and the size of this class shrinks over time. Further exploration reveals that nodes with *h*-index 0 are largely segregated from the rest of the networks and primarily collaborate with one another.

This explains why the highest values of StA are obtained with only two classes: because the lowest class (*h*-index = 0) is so strongly segregated from the rest of the network, the clearest division is between that class and everything else. With a larger number of classes, stratification also appears among nodes with *h*-index > 0 and increases over time. When the field is young, high *h*-index nodes collaborate with both medium and low *h*-index nodes, but such collaboration diversity wanes as the network gets older. One possible reason for this is that there are few high *h*-index researchers when the network is young, so high *h*-index must collaborate with lower *h*-index researchers. As more high *h*-index researchers become available, they prefer to work together and network gets more stratified.

**Figure 8.**
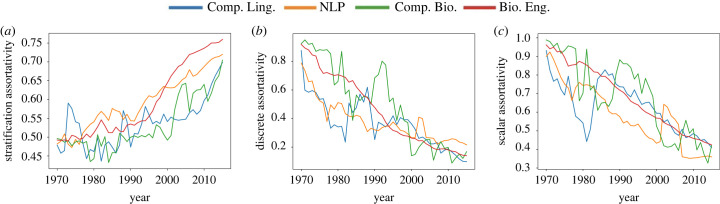
Metric comparison. (*a*) StA, (*b*) DAC and (*c*) SAC.

To better understand the stratification of these fields over time, we break down collaborations across pairs of classes. For simplicity, we use fixed class boundaries: in general, we find that it works well to partition nodes into four fixed classes of low-score nodes (*h*-index = 0), medium–low-score nodes (*h*-index ∈ {1, 2}), medium–high-score nodes (*h*-index ∈ {3, 4, 5, 6}) and high-score nodes (*h*-index > 6): this gives high stratification across time periods, while allowing for more granularity than only having two classes (analysis with three classes is very similar). The results for fixed classes are very close to the results of MaxStrat. We used fixed classes rather than the variable optimal classes for consistency of analysis across years (see figures 5*c* and 8*a*).

**Figure 7.**
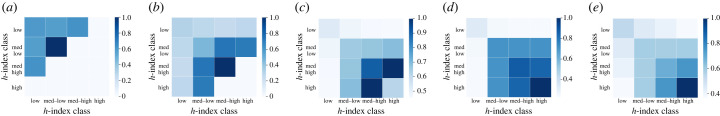
Collaborations from different *h*-index classes for NLP, normalized by degree (see description in text). (*a*) 1966–1975, (*b*) 1976–1985, (*c*) 1986–1995, (*d*) 1996–2005 and (*e*) 2006–2015.

Figure 7 shows a heatmap describing results in the NLP field (results for other fields are provided in electronic supplementary material, appendix). This plot shows the frequency of collaborations between classes of authors. Here, each cell (*c*_*i*_, *c*_*j*_) is the number of connections from class *c*_*i*_ to class *c*_*j*_, (|(*c*_*i*_, *c*_*j*_)|), normalized by the number of connections of the two classes (cell(*c*_*i*_, *c*_*j*_) = |(*c*_*i*_, *c*_*j*_)|/|*c*_*i*_| · |*c*_*j*_|, where |*c*_*i*_| is the number of connections where at least one side is in class *c*_*i*_). The *x*-axis and *y*-axis show the class of *h*-indices (like before, we divided the *h*-index scores into four classes). The results show that as network gets older, high-score nodes tend to collaborate with high-score nodes more.

#### 4.2.2. StA versus other assortativity metrics

Figure 8 compares StA with DAC and SAC using 5-year snapshots. For DAC and StA, we used the four fixed classes described in the previous section (for consistency of analysis: these boundaries are similar to those found by MaxStrat, but the optimal boundaries vary slightly from year to year); scalar assortativity was computed using the *h*-index scores directly. Unlike StA, DAC and SAC increase over time, and we examine each of these cases in turn below.

The primary driver behind the different behaviours of DAC and StA is that DAC is based on overall inter- versus intra-class connections, and so the sizes of classes (in terms of number of edges) weight the overall score, while StA is based on the average score of the classes and thus considers the impact of each class separately.

As an extreme case, consider a network in which there is only one class, and so all connections are intra-class connections. In this case, DAC is maximized (DAC = 1). However, this network is not highly stratified because there are no medium- or high-score nodes in the network (StA = 0.25).

We see a slightly less-extreme version of this phenomenon in the co-authorship networks. When the fields are young, the upper classes are very small compared with the lower classes. Most of the edges are thus between nodes in the lower classes. The classes are not segregated from one another; but the sheer size difference between the classes ensures that most edges are between nodes in the same (lower) class. Thus, DAC is high (because most edges are intra-class edges), but stratification is low, because the classes are not actually segregated from one another.

As the fields age, the upper classes fill out, and segregation increases. Because of this, StA increases. DAC, however, decreases, because as the class sizes become more balanced, the relative fraction of intra-class edges decreases more rapidly than the segregation between the classes increases.

Turning our attention to SAC, the differences between StA and SAC when fields are young is primarily due to class size. At this point, most researchers have very low *h*-indices and a large fraction of edges are between nodes with similar scores, so SAC is high. However, because the upper classes and lower classes are not segregated from one another, StA is low.

As the fields age, SAC decreases because the distribution and range of *h*-indices becomes wider, and so individuals may develop more diverse connections; for example, a node with an *h*-index of 30 might connect to a node with an *h*-index of 20. However, because these nodes are in the upper class, and so are considered similar by StA. Thus, while the *absolute* difference between scores of neighbours increases (on the whole), and so SAC decreases, the *class* difference between scores of neighbours decreases (on the whole), and so StA increases.

#### 4.2.3. StA and social mobility

**Figure 9.**
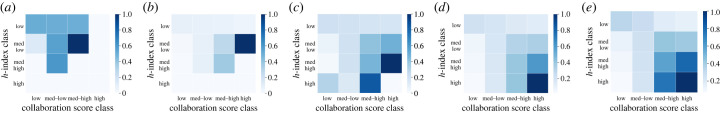
Relationship between entrance collaboration score of nodes and their *h*-index after 10 years for Biomedical Engineering. (*a*) 1966–1975, (*b*) 1976–1985, (*c*) 1986–1995, (*d*) 1996–2005, (*e*) 2006–2015.

A significant potential consequence of social stratification is a reduction of social mobility. We find that as networks age, the entrance point of new nodes has a larger effect on their trajectories through the field. For instance, figure 9 shows the relationship between entrance collaboration scores and *h*-index of researchers after 10 years in the Biomedical Engineering field (results in other fields are provided in electronic supplementary material, appendix).^4^ Cell (*c*_*i*_, *c*_*j*_) is the normalized number of authors with starting collaboration score from class *c*_*i*_ and *h*-index of class *c*_*j*_ after 10 years, (cell(*c*_*i*_, *c*_*j*_) = |(*c*_*i*_, *c*_*j*_)|/|*c*_*i*_| · |*c*_*j*_|, where |*c*_*i*_| is the number of authors in class *c*_*i*_). The *x*-axis shows the class of collaboration scores and the *y*-axis the class of *h*-indices (as before, each is divided into four tiers).

We observe that as the network ages, entrance point increasingly matters; and those who start their career by collaborating with high-score nodes become much more likely to achieve a high *h*-index themselves. A relationship between entry point and trajectory has been observed before [41]; but we demonstrate that this tendency tends to increase over time.

More analysis of StA on different numbers of classes/tiers is provided in electronic supplementary material, appendix.

### 4.3. Discussion

There are several major takeaways from our analysis:

(i) Stratification in scientific fields tends to increase over time. From figure 7, we see that this is due primarily to the increasing tendency of high *h*-index nodes to collaborate with other high *h*-index nodes.
(ii) Even as stratification increases, other assortativity metrics decrease: this occurs because even as nodes connect more within their own class, the diversity of their intra-class connections increases. This is true particularly for high *h*-index nodes.
(iii) As social stratification increases, social mobility decreases. This is a key observation, and has major implications for the career trajectory of new researchers. Simply put: when a field is new, a researcher’s trajectory is not strongly determined by their first few collaborators; but as the field ages, these early connections become increasingly important.

#### 4.3.1. Explaining stratification

We hypothesize that a major cause of stratification is related to access to collaborators. It is known that network proximity is important to researchers when forming new collaborations (e.g. through triadic closure [42]). When a field is young, and the collaboration network is less structured, a new researcher can find strong collaborators near them in the network: these collaborators can then help that new researcher boost her skills and profile. However, as the field ages, researchers with high *h*-index begin to find one another; and as they congregate, new researchers in other areas in the network have a much greater network distance from these older, successful researchers. This, in turn, makes it more difficult for those new researchers to join established projects, obtain mentoring from successful senior researchers, and so on. In contrast, researchers who happen to join the network in proximity to these high *h*-index researchers (e.g. the PhD students of such researchers) get a substantial head start.

The social science literature describes this phenomenon as *social distance*, which has long been understood as a cause of stratification [43]. In an extreme case, a stratified network might be divided into separate connected components corresponding to the various score intervals (tiers). This, naturally, has consequences for social mobility.

Outside of co-authorship networks, social distance is defined very broadly and can encompass characteristics like ethnicity, socioeconomic status, occupation, etc. A high social distance between classes suggests that individuals lack access—both directly and indirectly—to those in other classes. In a network setting, the simplest way to determine ‘access’ is by the existence of paths: if there is no path between two nodes, then by standard network evolution processes (e.g. triadic closure and the like), it is virtually impossible for them to connect in the future (this is not to say that they cannot connect; but if they do, it is probably because of processes external to the network topology).

To further investigate this hypothesis, we examined the properties of connected components in the co-authorship network. If connected components exhibit very different score-related characteristics, this is indicative of social distance driving the increase in stratification.

For each node *u* in the network, we compute a *collaboration score*, defined as the average *h*-index of the four highest-scoring collaborators of *u* (we only consider the top neighbours because access to higher-class individuals is more important than access to lower-class individuals for upwards social mobility). Note, importantly, that we are not simply using the scores of the nodes themselves: we are examining whether nodes in the various components have *access* to high *h*-index nodes.

Next, for each connected component, we compute the average of the collaboration scores of all nodes in that component. We refer to this as the *component score*. A higher value indicates that on average, nodes in the connected component have collaborations with high scoring nodes, while a lower average indicates that on average, nodes in the component lack connections to high scoring nodes. Note that a component with many low *h*-index nodes can still have a high component score, as long as those low *h*-index nodes have collaborations with high *h*-index nodes.

If social distance is indeed a driver of stratification, we expect these component scores to vary significantly across components: in some areas of the networks, nodes have access to high *h*-index nodes (high component scores), and in other areas, nodes do not (low component scores). Accordingly, we compute the standard deviation across the set of component scores. A low standard deviation indicates that nodes have similar collaboration patterns across different components of the network, and a high standard deviation indicates that the network has some components in which nodes tend to have higher collaboration scores and some components in which nodes tend to have lower collaboration scores. The former means that there are no major differences in social distance, suggesting that the network is not stratified; while the latter implies the converse. Note that we are not measuring stratification directly: rather, we are exploring whether there is a major difference in access to high *h*-index nodes in different regions of the network.

**Figure 10.**
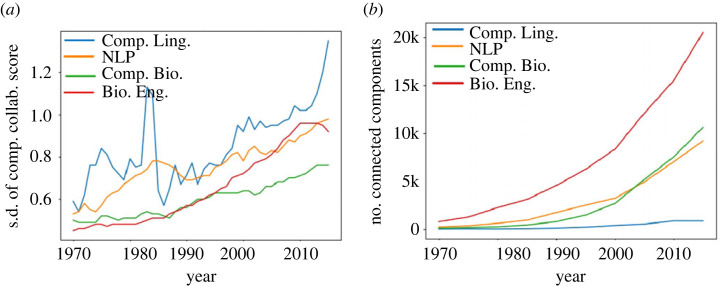
Standard deviation of average component collaboration scores over all components, and number of connected components. As the networks get older, standard deviation increases. (*a*) Standard deviation and (*b*) number of components.

Figure 10 shows these results. In all cases, as the network gets older, the number of components increases and the standard deviation across components increases. In other words, over time, the components show increasingly different behaviours from one another: in some, nodes have access to high scoring nodes, and in others, nodes do not. This matches the earlier stratification results exactly.

This analysis suggests a mechanism by which stratification in co-authorship networks could occur: if researchers have a preference for connecting to high *h*-index researchers, and there is a practical limitation on the number of researchers that one can collaborate with, then those researchers with a high *h*-index will tend to congregate with one another, leaving regions of the network devoid of such researchers. This then affects new researchers who enter the network in these areas, leading to decreased mobility.

Moreover, our study shows that the position of researchers in the network has a key role in their eventual success. In addition to our hypothesis, this can be due to different reasons such as privilege or discrimination. To explain this, we draw an analogy to results from *Stratification Economics*, a sub-field of economics that explains inter-group inequalities as caused, in part, by ‘uneven intergenerational transmission of resources and advantage’ [44–46]. Stratification economics examines differences between social classes, nations and regions, racial and ethnic groups, etc., in a competitive, cooperative or collaborative environment [44]. Studies in this area consider both the relative position of individuals within a social group as well as the absolute position of social groups, because both factors affect the lives, rewards and satisfaction of individuals [44].

Like the societies studied in stratification economics, scientific collaboration takes place in a cooperative and collaborative environment, and inequalities in access to resources and prestige can be ‘inherited’ through one’s collaborators. The citations that we use in this study are not directly inherited, but resources, prestige and status related to citations are unevenly transmitted across generations. Such factors can play a key role in facilitating the stratification process of collaboration networks, contributing to the development of hegemonical epistemologies that further reinforce stratification and, consequently, may lead to discrimination. For instance, certain research groups or universities have access to modern equipment, facilities and resources that gives their scholars an initial research advantage. These scholars may come to set the global research agenda, such that the work of researchers who lie beyond this network stratum becomes increasingly marginalized. For instance, Obeng-Odoom reviews research highlighting how the editorial boards of leading journals in economics are dominated by a small groups of (primarily white, Eurocentric) editors from mainstream economic departments [47]. As a result, mainstream journals tend to exclude research contributions that focus on topics that are of interest of African scholars. Although our study did not explore racial discrimination, it is clear from this example how discriminatory social consequences can arise from a network-based stratification process.

#### 4.3.2. Application and limitations of measuring stratification in other domains

While our analysis here has focused on co-authorship networks, the StA metric can be used to measure stratification in any system that has a network describing social or other relevant interactions, in which nodes are annotated with an appropriate score (the meaning of this score can vary by domain). It would be of particular interest to use it to study socioeconomic stratification: in particular, to gain another perspective on the extent to which personal and professional networks may affect class mobility, and to compare across different societies.

There are some important limitations in applying the StA metric. First, while the mathematics can be adapted to directed graphs, one should be careful in interpreting such results. On Twitter, for instance, there are many low-status individuals following a high-status individual, but this has very little relevance toward stratification as a whole, as it is very easy to follow someone, and reciprocity/consent are not required. Additionally, for similar reasons, it is important to consider the meaning of edge information. When studying online social media data, for instance, many platforms allow an effectively unlimited number of friends, and so high-status individuals may be willing to connect to low-status individuals; but such connections may not be meaningful. In such cases, the existence of communication or meaningful interaction may be more relevant than the existence of a ‘friendship’.

## 5. Conclusion and future work

In this paper, we proposed StA, a novel algorithm that measures network social stratification by evaluating the tendency of the network to be divided into ordered classes. Then, we performed a case study on several co-authorship networks and examined the evolution of these networks over time and showed that networks evolved into highly stratified states. In future work, we plan to study social stratification in other types of social networks, explore reasons behind stratification in different network types, and see how stratification can be prevented.

## Data Availability

The Microsoft Academic Graph (MAG) and underlying APIs were retired on December 2021 and the MAG dataset cannot be accessed any more per Microsoft’s policy. The datasets used in this paper are samples from MAG dataset. These samples contain co-authorship networks of researchers in four fields over 50 years. The whole co-authorship networks used in this paper are released and can be accessed in: https://github.com/SaraJalali/StratificationAssortativity/tree/main/Dataset. Please contact Zeinab S. Jalali (zsaghati@syr.edu) for further questions regarding the dataset. The data are provided in the electronic supplementary material [48].

## Endnotes

1 The maximum number *h* such that the author has at least *h* papers with at least *h* citations each.

2 We used MAG because it outperforms Google Scholar in terms of structure, functionality and richness of data [40].

3 Code and dataset can be accessed at: https://github.com/SaraJalali/StratificationAssortativity.

4 For authors in years 2012–2015 we examined their current *h*-index (2021).
